# Impact of dietary fenugreek sprouts and *β*-cyclodextrin on soft goat cheese

**DOI:** 10.1038/s41598-026-58674-w

**Published:** 2026-06-23

**Authors:** Marwa H. El-Gendy, Mahmoud Nassar, Alaa Emara Rabee, Osama Raef

**Affiliations:** 1 Animal Breeding Department, Desert Research Center, Ministry of Agriculture and Land Reclamation, Cairo, Egypt; 2 Animal and Poultry Nutrition Department, Desert Research Center, Ministry of Agriculture and Land Reclamation, Cairo, Egypt

**Keywords:** Goat milk, Fenugreek sprouts, *β*-cyclodextrin, Soft cheese, Texture, Biochemistry, Biotechnology, Health care

## Abstract

This study investigated the combined effects of fenugreek sprout feeding and *β*-cyclodextrin (*β*-CD) treatment on traditional soft goat cheese. Thirty-nine Hassani goats were divided into: control (C), F15 (15 g fenugreek sprouts dry/head/day), and F30 (30 g/head/day). Milk was processed with or without 0.5% *β*-CD. Fenugreek supplementation significantly enhanced milk production, F30 showing an approximately 60.6% increase in daily milk yield and improved fat and protein contents compared to C. In the cheese, F30 increased protein by 2.87%, while the addition of 0.5% *β*-CD (CF30) further increased protein by 5.0%, reduced cholesterol and triglycerides by 33.6 and 49.1%, respectively. Fenugreek supplementation improved the lipid profile, reducing total saturated fatty acids by 8.8% and increasing polyunsaturated fatty acids by 264.7% in F30. Textural analysis during storage showed hardness increasing by up to 343.8% in CF15, while springiness and cohesiveness declined. Microstructural confirmed a more homogeneous protein network in *β*-CD-treated samples. Sensory evaluation highlighted F15 and CF15 as the most acceptable treatments (scores of 8.8 and 8.2, respectively), whereas F30 and CF30 showed reduced acceptability. These findings demonstrated that moderate fenugreek supplementation (15 g/head/day), with or without *β*-CD, improved the nutritional quality, lipid profile, and structural characteristics of traditional soft goat cheese.

**Supplementary Information:**

The online version contains supplementary material available at 10.1038/s41598-026-58674-w.

## Introduction

Goat milk possesses distinctive biochemical properties, which differ significantly according to the breed of the animal and genetic polymorphism. Goat milk, in general, tends to have lower levels of α_s1_-casein and smaller average fat globule size compared to cow milk. These features are often associated a weaker gel network, higher syneresis, and lower firmness in fermented and coagulated dairy products^1^. These inherent challenges have encouraged research into technological interventions (e.g., heat processing, homogenization and addition of whey protein concentrates or polysaccharides) and nutritional approaches to achieving better product quality^2,3^.

Fenugreek (*Trigonella foenum-graecum*) seed supplements added to dairy goat rations have been reported to increase milk yield, modify fatty acids, and alter volatile compounds^4^. There is limited research on the use of fenugreek sprouts, although they contain extremely high amounts of bioactive components such as saponins and phenols^5^.

Technologically, *β*-cyclodextrin (*β*-CD) has come into focus to reduce cholesterol content via complexation of milk lipids and potentially affect the surface properties of fat globules, which in turn may affect whey–protein interaction and gel structure^6,7^**.** Additionally, it has been reported suppress off-flavors by sequestering volatile compounds^7^.

Despite numerous studies investigating the effect of fenugreek on milk production in ruminants, research examining the effects of fenugreek sprouts, which contain higher concentrations of bioactive compounds compared to the seeds, remains limited^5,8^. Furthermore, the combined effect of fenugreek feeding and *β*-CD treatment on the properties of soft cheese has not been sufficiently studied, representing a significant research gap addressed by this study. Addressing this gap offers a dual strategy to enhance goat cheese functionality, fortifying milk via nutrition and producing low cholesterol cheese with improved texture, providing value for both researchers exploring integrated approaches and the dairy industry seeking healthier functional products. Therefore, the objective of this research was to evaluate the impact of feeding different levels of fenugreek sprouts on the composition of goat milk, combined with *β*-CD treatment, and its effect on the rheological properties and microstructure of traditional soft cheese, to achieve a balanced nutritional and sensory quality.

## Material and methods

### Animals and materials

All experimental protocols and animal management procedures were formally approved by the Institutional Animal Care and Use Committee of the Desert Research Center, Egypt (Approval No. AN-5-2024), in accordance with the ARRIVE 2.0 guidelines and regulations, as well as the European Union standards for animal protection.

Samples for this three-month experiment were collected from 39 Hassani goats raised at the Shalateen Research Station, affiliated with the Desert Research Center, Wadi Hadarba, Halayeb, Red Sea Governorate, Egypt. The control group C was given a basal diet consisting of 60% concentrate feed mixture and 40% Egyptian clover hay (*Trifolium alexandrinum*), a widely used forage in Egypt characterized by its relatively high protein content and digestibility compared with other clover species. The detailed chemical composition and phytochemical profile of the Egyptian clover and the dietary ingredients are provided in Supplementary Table 1. Group F15 received a basal diet plus dry fenugreek sprouts (15 g/head/day), and Group F30 received a basal diet plus dry fenugreek sprouts (30 g/head/day). Despite the high concentrate level, no metabolic disturbances or negative effects on rumen fermentation were observed, as rumen pH values remained within the physiological range (5.96–6.15) as detailed in Supplementary Table 2. This indicated that the diet was well tolerated under the experimental conditions. Beta cyclodextrin (*β*-CD) was purchased from Wacker-Chemie Germany. FAR-M^®^ coagulant (CHY-MAX^®^ M, 1000 IMCU/mL, Chr. Hansen A/S, Hørsholm, Denmark) was used at a dosage of 0.05 mL/kg of milk. Pure salts of sodium chloride and calcium chloride were obtained from El-Nasr Company, Egypt.

### Starter cultures

CH-1 freeze-dried bacterial starter (*Lactobacillus delbrueckii* ssp. *bulgaricus* and *Streptococcus thermophilus*, Chr. Hansen, Denmark) were propagated in sterile skim milk and used at a 3% inoculation rate. Cultures were prepared 24 h prior to use.

### Milk sampling and analysis

Goats were milked twice daily, and individual milk yield was recorded. Fat, protein, lactose, ash and total solids of milk treatments were determined by ultrasonic milk analyzers (Master pro touch, Milkotester Ltd. Belovo, Bulgaria). Total cholesterol and triglyceride levels were determined by the spectrophotometric method using commercial enzymatic colorimetric kits (BioDiagnostic, Giza, Egypt). The chemical composition, total cholesterol and triglyceride of raw goat milk used in the experiment is shown in Table 1.

**Table 1:** Physicochemical properties of fresh goat milkobtained from goats fed different levels of fenugreek sprouts.

	C	F15	F30
Milk yield, g/d	643.67^a^ ± 81.07	743.75^a^ ± 123.3	1034.02^b^ ± 82.19
Fat	3.29^a^ ± 0.14	3.40^a^ ± 0.15	3.79^b^ ± 0.14
Protein	3.64 ± 0.04	3.71 ± 0.05	3.72 ± 0.42
Lactose	5.16 ± 0.07	5.49 ± 0.07	5.57 ± 0.06
Ash	0.98 ± 0.09	0.81 ± 0.09	0.82 ± 0.09
TS	13.07 ± 0.25	13.42 ± 0.27	13.89 ± 0.25
T. cholesterol (mg/dL)	81.20^a^ ± 0.16	80.10^b^ ± 0.09	78.60^c^ ± 0.09
Triglyceride (mg/dL)	20.71^a^ ± 0.74	17.36^b^ ± 0.21	9.79^c^ ± 0.61

^a-c^ Superscript lowercase letters within each row indicate statistically significant difference (*P* ≤ 0.05).

C = control (basal diet without supplement), F15 = (basal diet + 15 g/head/day fenugreek sprouts dry), and F30 = (basal diet + 30 g/head/day fenugreek sprouts dry).

### Pre-selection of β-CD level

A pre-processing level selection experiment was conducted to select the level of *β*-cyclodextrin at 0, 0.25, 0.50, 0.75 and 1.0% (w/v) prior to processing. The 0.50% treatment showed the optimal effect without a harmful effect on composition, texture, and microstructure. Therefore, 0.50% *β*-CD was selected as the optimal dosage for improving goat milk quality.

### Cheese manufacture and chemical analysis

Traditional soft cheese was produced from 36 kg of fresh goat milk obtain from goats fed 0, 15, and 30 g of dry fenugreek sprouts/head/day. Each milk group was divided into two subgroups: one processed without *β*-CD (control) designated as C, F15, and F30, respectively, and the other treated with 0.50% (w/v) *β*-CD designated as CC, CF15, and CF30, respectively. The milk from each subgroup was heated to 90 °C for 30 min to ensure full heat treatment and to induce partial denaturation of whey proteins, then rapidly cooled to 42 °C. CH-1 was inoculated at a rate of 3% and incubated for 30 min at 42 °C. FAR-M^®^ (0.05 mL/kg of milk) was then added at 42 °C to initiate the coagulation process until firm curds were obtained. The curds were then carefully transferred to perforated plastic molds lined with cheesecloths and left to drain overnight at 5 °C. The resulting cheese blocks were then placed in plastic containers and submerged in their own whey, to which salt (2% w/w) had been added to maintain a soft texture. The containers were tightly sealed to ensure a high relative humidity (~ 85%) and stored for a 3-week ripening period at 5 ± 1 °C. Chemical composition, total fatty acids, and volatile fatty acids were determined in the traditional fresh cheese samples. To monitor the changes during storage, textural properties, sensory evaluation, and microstructure analysis were performed on both fresh cheese (day 1) and after the 3 weeks ripening period. Moisture, total solids, protein and Ash of cheese samples were determined according to^9^. Fat content was determined by Gerber’s method. Fat in dry matter (Fat/DM) was calculated according to the equation: Fat/DM (%) = (Fat content × 100)/(100−Moisture content). Total cholesterol and triglyceride levels were determined spectrophotometrically as previously described for milk.

### Extraction and identification of volatile and fatty acids

Fatty acids were analyzed after lipid extraction and methylation into fatty acid methyl esters following^10^. The composition of long-chain and volatile fatty acids (VFAs) was determined using gas chromatography system (Agilent GC 8890, Agilent Technologies, USA) equipped with a Flame Ionization Detector (FID) and a Zebron ZB-FAME capillary column (60 m × 0.25 mm ID × 0.20 μm film thickness; Phenomenex, Torrance, CA, USA). Injector and detector temperatures were set at 250 and 285 °C, respectively, and results were expressed as a percentage of total fatty acids. This setup allowed for the precise quantification of a wide range of fatty acids, from C4:0 to C22:0. Volatile fatty acids (VFAs; C2—C10) were quantified using a modified method of^11^. This approach allowed simultaneous evaluation of both long- and short-chain fatty acids to assess nutritional and flavor-related lipid changes due to fenugreek feeding and *β*-CD treatment.

### Texture profile analysis

The textural properties of traditional soft goat cheese were evaluated using a Texture Analyzer (Model TL-Pro, Lloyd Instruments, UK) following the standard double-compression TPA test described by^12^. Cheese cubes (2 × 2 × 2 cm) were equilibrated at 20 ± 1 °C and compressed twice to 50% of their original height at 1 mm s⁻^1^ with a 5 s interval. Primary texture parameters (hardness, adhesiveness, cohesiveness, springiness, gumminess, chewiness, and modulus, which reflects the stiffness or resistance of the cheese to deformation) were automatically calculated. Each treatment and storage period (fresh and 3 weeks) was analyzed in triplicate.

### Microstructure analysis

The microstructure of traditional fresh cheese samples was examined by scanning electron microscopy following^13^. Small cheese cubes were fixed in glutaraldehyde, dehydrated in graded ethanol, and gold-coated before imaging. Micrographs were evaluated for compactness, pore size, and fat distribution to compare the effects of fenugreek supplementation and *β*-CD treatment on the protein–fat matrix organization.

### Sensory evaluation

Sensory evaluation was carried out by a trained panel of 10 members from the Dairy unit, Animal Breeding Department, Desert Research Center. The sensory evaluation protocol followed the ethical guidelines for human food testing; all panelists were informed about the nature of the samples and provided their informed consent prior to participation. All cheese samples were prepared using food grade ingredients under hygienic conditions. Cheese samples for fresh and end storage were evaluated for appearance, color, texture, flavor, and overall acceptability using a 9-point hedonic scale (1 = dislike extremely, 9 = like extremely). Additionally, the panelists were asked to provide a descriptive evaluation for any perceived off-flavors, including rancidity, bitterness, or soapy tastes, to monitor the impact of fenugreek and *β*-CD treatment on the sensory profile. Each treatment was presented in randomized order under white light at room temperature (20 ± 1 °C).

### Statistical analysis

Data were analyzed using one- and two-way ANOVA followed by Duncan’s multiple range test at *P* ≤ 0.05 using SAS software (version 12.1; SAS Institute Inc., Cary, NC, USA)^14^.

## Results and discussion

### Chemical composition of traditional soft goat cheese

The chemical composition results in Table 2 reveal significant improvements in the nutritional profile of traditional soft goat cheese. Fenugreek supplementation led to increases in total solids (from 32.8% in C to 33.6% in F30) and protein contents (from 13.9 to 14.3%), representing a 2.87% increase, which reflects improved nutritional quality of the milk^4^. The slightly higher moisture content in *β*-CD treated samples (e.g., 67.6% in CC) compared to untreated ones (67.2% in C) suggests that *β*-CD interacts with the protein–fat matrix, enhancing its water-holding capacity through inclusion complexes within the casein network^7^. The elevated fat content in fenugreek-fed treatments reflects the lipid-modifying effects of bioactive compounds in fenugreek, particularly saponins, which can alter rumen fermentation and enhance lipid mobilization and secretion into milk^15^.

**Table 2:** Chemical composition, cholesterol, and triglyceride levels of traditional soft goat cheese as affected by dietary fenugreek sprouts and *β*-CD treatment.

Parameter	C	F15	F30	CC	CF15	CF30	± SE
Moisture %	67.2^b^	66.8^b^	66.4^b^	67.6^a^	67.3^a^	66.9^a^	0.35
Total Solids%	32.8^b^	33.2^b^	33.6^a^	32.4 ^b^	32.7^b^	33.1^a^	0.35
Fat%	19.1^ab^	19.5^a^	19.9^a^	18.8^b^	19.1^ab^	19.4^a^	0.22
Protein%	13.9^b^	14.1^ab^	14.3^a^	13.9^b^	14.2^a^	14.6^a^	0.18
Ash%	2.05^b^	2.10^ab^	2.15^a^	2.10^b^	2.20^a^	2.25^a^	0.06
Fat/DM%	58.2^b^	58.7^ab^	59.2^a^	58.1^b^	58.4^ab^	58.7^a^	1.21
T. Cholesterol (mg/dL)	62.5^a^	59.2^a^	55.8^b^	47.7^b^	44.8^b^	41.5^c^	1.92
Triglycerides (mg/dL)	21.8^a^	18.4^a^	15.9^b^	15.6^b^	12.8^b^	11.1^c^	0.95

^a-c^ Superscript lowercase letters within each row indicate significant difference (*P* ≤ 0.05).

C, F15 and F30 = Soft cheese produced from milk of goats fed 0, 15, and 30 g/head/day fenugreek sprouts, respectively (without *β*-CD).

CC, CF15 and CF30 = Soft cheese produced from the same milk groups and treated with 0.50% (w/v) *β*-CD during processing.

Fat content was significantly (*P* ≤ 0.05) affected by *β*-CD treatment (Table 2). A consistent reduction in fat percentage was observed in *β*-CD treated samples (e.g., from 19.1% in C to 18.8% in CC; ≈1.57% decrease). This reduction may be attributed to the selective removal of certain lipid fractions during complex formation^16,17^. However, the fat in dry matter (58.1–59.2%) remained stable, indicating efficient fat recovery. Protein content reached its highest value in CF30 (14.6%; ≈ 5.03% increase) over the control, likely due to better rumen nitrogen utilization from fenugreek bioactives and the stabilizing effect of *β*-CD^18^.

Notably, the addition of 0.5% *β*-CD significantly (*P* ≤ 0.05) reduced cholesterol and triglyceride contents. Cholesterol decreased from 62.5 mg/dL in C to 41.5 mg/dL in CF30 (≈33.6% reduction) while triglycerides declined from 21.8 mg/dL to 11.1 mg/dL (≈49.1% reduction). This reduction may be attributed to *β*-CD’s ability to entrap cholesterol molecules within its hydrophobic cavity^19,20^. Complementarily, fenugreek saponins further lowered cholesterol by interfering with its absorption^21^, while triglycerides decreased due to fenugreek’s influence on lipid metabolism and *β*-CD’s binding capacity^18^. The combined strategy resulted in improved protein content, reduced cholesterol and triglycerides, and maintained fat recovery, supporting the development of nutritionally enhanced traditional soft goat cheese while preserving its compositional balance.

### Fatty acid profile of traditional soft goat cheese

Table 3 shows the fatty acid composition of traditional soft goat cheese, revealing clear differences depending on the level of fenugreek sprout feeding and the use of *β*-CD during processing. Fenugreek supplementation resulted in a reduction in total saturated fatty acids (ΣSFA) from 71.71% in C to 65.40% in F30 (≈8.8% decrease). This decrease may be associated with reductions in major saturated fatty acids such as C16:0 (from 27.67 to 23.78%) and C18:0 (from 7.49 to 5.20%). In contrast, total polyunsaturated fatty acids (ΣPUFA) increased markedly from 3.22% in C to 11.74% in F30 (≈264.7% increase). For instance, linoleic acid (C18:2) increased from 2.19 to 7.09% (≈223.7%), while linolenic acid (C18:3) rose from 1.02 to 4.65% (≈355.9%) in the F30 treatment. In addition, the n-6/n-3 ratio improved from 2.15 in C to 1.52 in F30, indicating enhanced nutritional quality of the milk fat. These changes may be attributed to the bioactive compounds in fenugreek, particularly saponins, which are known to modify rumen fermentation patterns by suppressing protozoal populations and altering lipid metabolism pathways, thereby increasing the outflow of unsaturated fatty acids (USFA) from the rumen^22^. These findings are consistent with those reported by Akbağ et al.^4^, who demonstrated that fenugreek seed supplementation effectively modifies the milk fat composition in goats.

**Table 3:** Fatty acid profile (%) of traditional soft goat cheese produced with dietary fenugreek sprouts and *β*-CD treatment.

Fatty acid	C	F15	F30	CC	CF15	CF30	± SE
C4:0	3.20	4.20	4.60	3.05	4.16	4.50	0.20
C6:0	2.10	1.68	1.35	2.06	1.50	1.30	0.10
C8:0	9.19	8.09	8.60	9.10	7.97	8.50	0.42
C10:0	12.49	10.37	11.16	12.10	10.00	11.12	0.55
C12:0	3.10	2.59	2.10	2.97	2.50	2.00	0.13
C14:0	4.11	3.60	3.10	4.09	3.50	3.00	0.18
C14:1	0.55	1.55	1.97	0.50	1.50	2.00	0.09
C15:0	1.10	3.07	3.60	1.09	2.96	3.50	0.16
C16:0	27.67	25.94	23.78	26.98	24.82	22.86	1.30
C16:1	2.10	3.10	2.60	2.06	3.00	2.50	0.14
C18:0	7.49	6.50	5.20	6.95	6.00	5.00	0.35
C18:1	22.43	20.19	18.42	23.26	21.02	19.19	1.05
C18:2	2.19	6.24	7.09	2.55	7.00	7.96	0.38
C18:3	1.02	2.46	4.65	1.03	3.00	4.88	0.22
C20:0	0.62	1.00	1.60	0.50	1.00	1.50	0.06
C22:0	0.64	0.09	0.30	0.56	0.02	0.20	0.03
Σ SFA	71.71	67.14	65.40	69.44	64.40	63.48	1.34
Σ MUFA	25.07	24.83	22.99	25.81	25.56	23.69	0.50
Σ PUFA	3.22	8.70	11.74	3.58	10.00	12.84	0.55
n-6/n-3 ratio	2.15	2.54	1.52	2.49	2.33	1.63	

C, F15 and F30 = Soft cheese produced from milk of goats fed 0, 15, and 30 g/head/day fenugreek sprouts, respectively (without *β*-CD).

CC, CF15 and CF30 = Soft cheese produced from the same milk groups and treated with 0.50% (w/v) *β*-CD during processing.

Σ SFA = Total saturated fatty acids, Σ MUFA = Total monounsaturated fatty acids.

Σ PUFA = Total polyunsaturated fatty acids; n-6/n-3 ratio = Ratio of omega-6 to omega-3 fatty acids.

Regarding *β*-CD treatment, only minor variations were observed in fatty acid distribution (e.g., ΣSFA decreased from 69.44% in CC to 63.48% in CF30), suggesting that *β*-CD primarily influences lipid organization rather than fatty acid synthesis. Its contribution to cheese quality may be attributed to its ability to form inclusion complexes with lipid molecules, including free fatty acids and cholesterol^19^, thereby reducing their interference with the casein matrix and promoting a more homogeneous protein network^7,18,23^. In the present study, the combined nutritional technological approach enhanced the fatty acid profile by reducing SFA and increasing PUFA content, while *β*-CD contributed mainly to structural stabilization, resulting in improved nutritional and technological quality of the final product.

### Volatile fatty acid composition of traditional soft goat cheese

Analysis of Volatile fatty acid** (**VFA) in traditional soft goat cheese showed clear differences depending on both the level of fenugreek feeding and the use of *β*-CD during processing (Table 4). Fenugreek feeding resulted in a marked increase in short-chain fatty acids, butyric acid (C4), which increased from 0.40% in C to 0.59% in F30 (≈47.5% increase), and valeric acid (C5), which rose from 72.00 to 82.00% (≈13.9% increase). Acetic acid (C2) also increased from 0.25 to 0.60% (≈140% increase) with increasing fenugreek levels. In contrast, propionic acid (C3) decreased substantially from 22.00% in C to 10.20% in F30 (≈53.6% reduction), indicating a shift toward butyrate- and valerate-producing fermentation pathways in the rumen. These findings are consistent with previous studies reporting that fenugreek supplementation alters ruminal microbial activity and VFA production patterns^4,24^.

**Table 4:** Volatile fatty acid (VFA) concentrations of traditional soft goat cheese influenced dietary fenugreek sprouts and *β*-CD treatment.

VFAs	C	F15	F30	CC	CF15	CF30	± SE
C2	0.25	0.45	0.60	0.22	0.41	0.55	0.03
C3	22.00	15.50	10.20	20.57	14.68	9.50	1.33
C4	0.40	0.55	0.59	0.41	0.56	0.70	0.04
C5	72.00	77.20	82.00	73.40	78.35	82.90	3.00
C6	1.60	1.80	2.00	1.55	1.70	1.90	0.13
C8	2.90	3.20	3.50	2.85	3.10	3.40	0.22
C10	0.85	1.00	1.10	0.82	0.95	1.05	0.08

C, F15 and F30 = Soft cheese produced from milk of goats fed 0, 15, and 30 g/head/day fenugreek sprouts, respectively (without *β*-CD).

CC, CF15 and CF30 = Soft cheese produced from the same milk groups and treated with 0.50% (w/v) *β*-CD during processing.

Regarding *β*-CD treatment, the formation of inclusion complexes led to slightly lower concentrations of some volatile fractions (e.g., C3 decreased from 22.00% in C to 20.57% in CC, and from 10.20% in F30 to 9.50% in CF30). This may suggest improved protection of fatty acids against oxidation and a more balanced flavor profile^18,23^. Overall, increasing fenugreek levels enhanced VFA production, while *β*-CD treatment was associated with slightly lower VFA concentrations. This combined effect may contribute to a more balanced flavor profile and improved oxidative stability in the resulting cheese.

### Texture profile analysis of traditional soft goat cheese

The Texture profile analysis data indicate significant effects of both *β*-CD treatment and fenugreek supplementation on the mechanical behavior of traditional soft goat cheese (Table 5). Hardness increased significantly after 3 weeks in all treatments, for instance, in the control (C), hardness increased from 1.79 to 2.60 N (≈45.3% increase), while in CF15 it increased from 0.80 to 3.55 N (≈343.8% increase). Similarly, the modulus values showed a consistent upward trend across all treatments during ripening, with CF15 exhibiting the most pronounced increase from 0.08 to 0.42 N/mm^2^ (≈ 425% increase) by the end of storage. This rise in hardness and modulus may be attributed to moisture loss and progressive tightening of the protein matrix during storage, as whey migration and protein–protein interactions continue post-manufacture^25^. Furthermore, *β*-CD-treated samples (CC, CF15, CF30) exhibited higher hardness and modulus values after storage (3.20 to 3.55 N and 0.35 to 0.42 N/mm^2^, respectively) compared to non-treated counterparts (2.56 to 2.84 N, 0.28 to 0.33 N/mm^2^, ≈20 to 25% higher), indicating the formation of a more compact and cohesive structure. This effect may be related to the ability of *β*-CD to form inclusion complexes with free lipids and cholesterol, thereby improving protein–fat interactions within the curd matrix^26^.

**Table 5:** Changes in texture profile analysis parameters of traditional soft goat cheese during 3 weeks of storage (5 ± 1 °C) as affected dietary fenugreek sprouts and *β*-CD treatment.

Storage	Treatment	Hardness (N)	Adhesiveness (MJ)	Cohesiveness (ratio)	Springiness (mm)	Gumminess (N)	Chewiness (mJ)	Modulus (mm)
Fresh	C	1.79 ± 0.13^Bb^	0.21 ± 0.01^Abc^	0.62 ± 0.04^Ab^	6.92 ± 0.48 ^Aa^	1.35 ± 0.09 ^Aa^	10.95 ± 0.77 ^Aa^	0.95 ± 0.07 ^Aa^
	F15	0.68 ± 0.05^Bc^	0.29 ± 0.02^Ab^	0.69 ± 0.05^Aab^	6.93 ± 0.49 ^Aa^	0.59 ± 0.04 ^Ab^	4.93 ± 0.35 ^Ab^	0.95 ± 0.07 ^Aa^
	F30	0.77 ± 0.05^Bc^	0.37 ± 0.03^Aa^	0.46 ± 0.03^Ac^	6.93 ± 0.49 ^Aa^	0.45 ± 0.03 ^Ab^	3.48 ± 0.24 ^Ab^	0.95 ± 0.07 ^Aa^
3 weeks	C	2.60 ± 0.18^Ab^	0.15 ± 0.01^Bc^	0.49 ± 0.03^Bb^	0.71 ± 0.05 ^Bb^	0.41 ± 0.03 ^Bb^	1.07 ± 0.07 ^Bb^	0.50 ± 0.04 ^Ba^
	F15	2.84 ± 0.20^Ab^	0.24 ± 0.02^Bb^	0.54 ± 0.04^Bb^	0.59 ± 0.04 ^Bb^	0.48 ± 0.03 ^Bb^	1.08 ± 0.08 ^Bb^	0.43 ± 0.03 ^Ba^
	F30	2.56 ± 0.18^Ab^	0.33 ± 0.02^Ba^	0.42 ± 0.03^Bc^	0.12 ± 0.01 ^Bc^	0.39 ± 0.03 ^Bb^	0.16 ± 0.01 ^Bc^	0.25 ± 0.02 ^Bb^
Fresh	CC	2.10 ± 0.15^Ba^	0.19 ± 0.01^Ac^	0.73 ± 0.05^Aa^	6.59 ± 0.46 ^Aa^	1.50 ± 0.11^Aa^	9.95 ± 0.70 ^Aa^	0.83 ± 0.06 ^Ab^
	CF15	0.80 ± 0.06^Bc^	0.25 ± 0.02^Ab^	0.82 ± 0.06^Aa^	6.60 ± 0.46 ^Aa^	0.65 ± 0.05 ^Ab^	4.48 ± 0.31 ^Ab^	0.83 ± 0.06 ^Ab^
	CF30	0.90 ± 0.06^Bc^	0.32 ± 0.02^Aa^	0.54 ± 0.04^Ab^	6.60 ± 0.46^Aa^	0.50 ± 0.04^Ab^	3.16 ± 0.22 ^Ab^	0.83 ± 0.06 ^Ab^
3 weeks	CC	3.25 ± 0.23 ^Aa^	0.12 ± 0.01 ^Bc^	0.60 ± 0.04 ^Ba^	0.59 ± 0.04 ^Bb^	0.45 ± 0.03 ^Bb^	0.89 ± 0.06^Bb^	0.21 ± 0.02 ^Bb^
	CF15	3.55 ± 0.25 ^Aa^	0.19 ± 0.01 ^Bb^	0.65 ± 0.05 ^Ba^	0.49 ± 0.03 ^Bb^	0.53 ± 0.04 ^Ba^	0.91 ± 0.06 ^Bb^	0.14 ± 0.01 ^Bc^
	CF30	3.20 ± 0.22 ^Aa^	0.26 ± 0.02 ^Ba^	0.50 ± 0.04 ^Bb^	0.10 ± 0.01 ^Bc^	0.44 ± 0.03 ^Bb^	0.14 ± 0.01 ^Bc^	0.12 ± 0.01^Bc^

^A–B^ Uppercase letters within the same column for each parameter indicate significant differences (P ≤ 0.05) due to storage period.

^a–c^ Lowercase letters within the same column for each parameter indicate significant differences (P ≤ 0.05) among dietary treatment and *β*-CD levels.

C, F15 and F30 = Soft cheese produced from milk of goats fed 0, 15, and 30 g/head/day fenugreek sprouts, respectively (without *β*-CD).

CC, CF15 and CF30 = Soft cheese produced from the same milk groups and treated with 0.50% (w/v) *β*-CD during processing.

Adhesiveness and springiness decreased during storage; for instance, adhesiveness in C it declined from 0.213 to 0.151 mJ (≈29.1% reduction), consistent with reduced surface moisture. Similarly, springiness showed a marked decrease, particularly in F30 (from 6.93 to 0.12 mm, ≈98% reduction) and CF30 (from 6.60 to 0.10 mm, ≈98.5% reduction), indicating pronounced matrix stiffening at higher fenugreek levels. This behavior may be associated with interaction between fenugreek polysaccharides and saponins with casein micelles, leading to reduced gel porosity and elastic recovery^27^.

Gumminess and Chewiness, both parameters showed a sharp decline during storage despite the increase in hardness. For example, gumminess in the control (C) decreased from 1.35 to 0.41 N (≈69.6% reduction), while chewiness declined from 10.95 to 1.07 mJ (≈90.2% reduction). Similar trends were observed in *β*-CD-treated samples, with chewiness in CF30 decreasing from 3.16 to 0.14 mJ (≈95.6% reduction). This reduction reflects the combined influence of decreased cohesiveness and springiness despite the increase in hardness. Since gumminess is a function of hardness × cohesiveness and chewiness is derived from gumminess × springiness, the substantial loss in elasticity (springiness) and internal bonding (cohesiveness) during storage plays a dominant role in lowering these parameters. These changes may be attributed to structural rearrangements within the protein matrix, including moisture loss, increased protein aggregation, and reduced network flexibility over time^27,28^. Overall, the moderate fenugreek level (CF15) maintained a more balanced textural profile after storage, whereas higher levels (CF30) led to an over-compacted and less elastic structure.

### Microstructure of traditional soft goat cheese

**Fig. 1:**
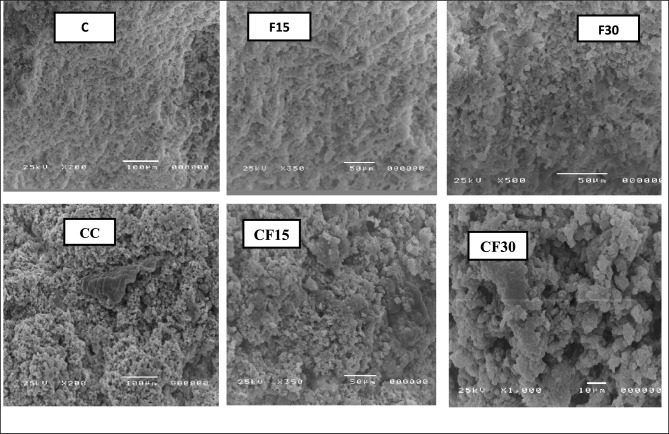
Scanning electron micrographs (SEM) showing the microstructure of soft goat cheese as influenced by dietary fenugreek sprouts and *β*-CD treatment. C, F15, and F30 = cheese samples without *β*-CD; CC, CF15, and CF30 = cheese samples treated with 0.5% *β*-CD. Magnification levels and scale bars are as follows: (C, CC) at × 200, scale bar = 100 µm; (F15, CF15) at × 350, scale bar = 50 µm; (F30) at × 500, scale bar = 50 µm; and (CF30) at × 1000, scale bar = 10 µm.

Scanning Electron Microscope revealed distinct structural modifications in traditional soft goat cheese resulting from fenugreek sprout supplementation and *β*-CD treatment (Fig. 1). It should be noted that while micrographs were captured at different magnifications, clear trends in matrix organization were observed. In treatments without *β*-CD, the protein matrix appeared coarse, less compact, and characterized by irregular pores and visible fat globules, particularly in C, which is consistent with the typical structure of soft cheeses lacking stabilizing agents^29^. The F15 sample showed a slightly more continuous structure yet still exhibited non-uniform porosity and larger fat voids within the protein network. At F30, the matrix appeared denser with partial aggregation of protein strands, which may be associated with the influence of fenugreek components on curd structure^30^.

Conversely, the *β*-CD-treated cheeses exhibited a fine-grained and homogeneous network, with smaller and more evenly distributed fat globules within the protein matrix. These observations are consistent with previous studies on *β*-CD-treated cheese systems^7,26,28^. The CF15 treatment showed a relatively dense and continuous matrix with more uniform structural distribution, whereas CF30 appeared more compact with reduced porosity^27^. Overall, *β*-CD supplementation was associated with improved microstructural uniformity and matrix compactness, which is consistent with the observed changes in textural properties and the reported ability of *β*-CD to reduce porosity and enhance textural stability in various dairy systems^26,28^.

### Sensory evaluation of traditional soft goat cheese

Table 6 presents the sensory evaluation scores of traditional soft goat cheese as affected by fenugreek supplementation and *β*-CD treatment during storage. In fresh cheese (Day 1), treatment F15 achieved the highest overall acceptability score (8.8), compared with 8.2 in the control (C), representing an increase of approximately 7.3%. This improvement was consistent across flavor (8.8 vs. 8.3) and texture (8.9 vs. 8.4), which may be attributed to the development of desirable aromatic compounds and improved microstructure^4,18^. In contrast, a significant (*P* ≤ 0.05) decreasing trend in acceptability was observed with higher fenugreek inclusion; specifically, CF30 received the lowest scores at Day 1 (Flavor: 6.8, Texture: 7.2), representing a decrease of approximately 18–20% compared to C. This reduction may be associated with the higher fenugreek level and its influence on flavor balance^24,30^ and was also associated with weaker textural attributes (decreased hardness and cohesiveness) in Table 5. Notably, no pronounced rancidity related off-flavors were perceived by the panelists, indicating that the lower scores were mainly due to increased herbal intensity rather than lipid oxidation.

**Table 6:** Sensory evaluation scores of traditional soft goat cheese at day 1 and after 3 weeks of storage as affected by dietary fenugreek sprouts and *β*-CD treatment.

Treatment	C	F15	F30	CC	CF15	CF30	± SE
Fresh							
Appearance	8.0^Aa^	8.6^Aa^	7.8^Aa^	7.8^Aa^	8.3^Aa^	7.0^Bb^	0.210
Color	8.1^Aa^	8.7^Aa^	8.0^Aa^	7.7^Aa^	8.2^Aa^	7.1^Bb^	0.257
Flavor	8.3^Aa^	8.8^Aa^	7.9^Aa^	7.5^Aa^	8.0^Aa^	6.8^Bb^	0.237
Texture	8.4^Aa^	8.9^Aa^	8.2^Aa^	7.6^Aa^	8.4^Aa^	7.2^Bb^	0.238
Overall	8.2^Aa^	8.8^Aa^	8.0^Aa^	7.7^Aa^	8.2^Aa^	7.0^Bb^	0.228
3 weeks							
Appearance	7.7^Ba^	8.3^Aa^	7.5^Ba^	7.2^Ba^	7.8^Ba^	6.5^Cb^	0.203
Color	7.6^Ba^	8.4^Aa^	7.8^Ba^	7.0^Ba^	7.6^Ba^	6.7^Cb^	0.215
Flavor	7.5^Ba^	8.5^Aa^	7.7^Ba^	6.8^Bb^	7.4^Ba^	6.3^Cc^	0.233
Texture	7.6^Ba^	8.6^Aa^	7.8^Ba^	7.0^Bb^	7.7^Ba^	6.6^Cc^	0.228
Overall	7.6^Ba^	8.5^Aa^	7.7^Ba^	6.9^Bb^	7.6^Ba^	6.4^Cc^	0.225

^a–c^: Lowercase letters within each column indicate significant differences (*P* ≤ 0.05) among fenugreek levels.

^A–C^: Uppercase letters indicate significant differences (*P* ≤ 0.05) between *β*-CD treatments and storage times.

C, F15 and F30 = Soft cheese produced from milk of goats fed 0, 15, and 30 g/head/day fenugreek sprouts, respectively (without *β*-CD).

CC, CF15 and CF30 = Soft cheese produced from the same milk groups and treated with 0.50% (w/v) *β*-CD during processing.

After three weeks of storage, all sensory attributes decreased significantly (*P* ≤ 0.05). However, F15 maintained the highest overall score (8.5), followed by CF15 (7.6), whereas CF30 showed the lowest value (6.4), representing a reduction of approximately 24.7% compared with F15. Flavor scores decreased slightly in F15 (from 8.8 to 8.5; ≈3.4%), while a greater decline was observed in CF30 (from 6.8 to 6.3; ≈7.4%). This indicates that moderate fenugreek inclusion supports better sensory retention and may contribute to better resistance to oxidative changes during storage^19,23^. While control samples (C and CC) had high sensory scores (e.g., 8.2 and 7.7 for overall acceptability), they showed greater susceptibility to sensory deterioration over time. In contrast, treatments F15 and CF15 balanced these sensory characteristics with nutritional improvements, specifically the increased PUFA content and reduced cholesterol and triglyceride levels^4,23^. The lower scores observed in F30 and CF30 are therefore more likely associated with increased herbal intensity and slight bitterness related to higher fenugreek inclusion rather than lipid oxidation^30^. Overall, moderate fenugreek supplementation (15 g/head/day), with or without *β*-CD, was associated with improved sensory acceptability, storage stability and nutritional quality supporting its suitability as the optimal treatment level.

While this study provides valuable insights into the combined effects of fenugreek sprout feeding and *β*-CD treatment on goat cheese quality, several limitations should be acknowledged. First, the experiment was conducted using a single goat breed (Hassani) under specific regional conditions, which may limit the generalizability of the findings to other breeds or production systems. Second, the ripening period was restricted to three weeks; further studies focused on the stability of bioactive compounds and oxidative markers towards the end of the storage period would be beneficial. Finally, although sensory evaluation was performed using a trained panel, further large-scale consumer acceptance testing would be beneficial to confirm the broader acceptability of the most favorable treatments under market conditions.

## Conclusion

The present study indicated that fenugreek supplementation and *β*-cyclodextrin (*β*-CD) treatment influenced the quality characteristics of traditional soft goat cheese. The nutritional value of the cheese was enhanced by fenugreek supplementation (C to F30), as indicated by an increase in the total solids from 32.8 to 33.6%, protein content from 13.9 to 14.3%, and enhancing the fatty acid profile by reducing total saturated fatty acids (ΣSFA) from 71.71 to 65.40% ((≈8.8% decrease), while increasing of total polyunsaturated fatty acids (ΣPUFA) from 3.22 to 11.74%. Moreover, the effect of *β*-CD treatment (C to CC) contributed to improving lipid related attributes, resulting in reducing the fat content (≈1.6%), as well as lowering the cholesterol content (≈23.7%) and triglycerides (≈28.4%). Textural properties changed during storage, indicating the strengthening and compaction of the protein matrix, consistent with the observed microstructural variations. The sensory evaluation showed that the highest overall acceptability of traditional fresh cheese was obtained with moderate fenugreek supplementation levels (F15), with scores of 8.8 compared with the control of 8.2 (≈7.3%), while after storage scores of 8.5 to 7.6 (≈11.8%). However, the highest fenugreek supplementation (F30 and CF30) produced lower sensory scores. No pronounced rancidity related off-flavors were associated with higher fenugreek supplementation. In conclusion, moderate fenugreek supplementation (15 g/head/day), combined with or without *β*-CD treatment, was associated with improved nutritional quality, enhanced fatty acid profile, and better sensory acceptability of traditional soft goat cheese under the experimental conditions.

## Supplementary Information

Supplementary Information.

## Data Availability

The raw data of this study is available by the corresponding author upon request.
