# Supplementary material for: Impact of dietary fenugreek sprouts and β-cyclodextrin on soft goat cheese
Source: Sci Rep. 2026 Jun 23;16:19527. doi: 10.1038/s41598-026-58674-w (PMC13291265; doi:10.1038/s41598-026-58674-w)
Supplement: Supplementary file 1 — Supplementary Information. [file 41598_2026_58674_MOESM1_ESM.docx]

**Supplementary Table 1: Proximate chemical composition, total phytochemicals, and polyphenolic profile of dietary ingredients (on dry matter basis).**

|  | **Fenugreek sprouts** | **CFM** | **Egyptian clover** |  |
| --- | --- | --- | --- | --- |
| **Proximate chemical composition, %** | | | | |
| DM | 33.50 | 92.00 | 90.00 |  |
| EE | 13.03 | 8.52 | 6.21 |  |
| CP | 36.50 | 14.00 | 15.00 |  |
| CF | 12.00 | 7.30 | 22.00 |  |
| NDF | 34.00 | 30.00 | 55.00 |  |
| Ash | 4.12 | 4.09 | 16.00 |  |
| NFE | 34.35 | 66.09 | 40.79 |  |
| **Phytochemicals, %** | | | | |
| Phenols | 0.15 | 0.05 | 0.09 |  |
| Flavonoids | 0.41 | 0.40 | 0.31 |  |
| Tannins | 0.11 | 0.17 | 0.18 |  |
| **Polyphenols profile, µg/g** | | | | |
| Gallic acid | 40.75 | 1.50 | 00 |  |
| Chlorogenic acid | 41.54 | 0.10 | 00 |  |
| Ellagic acid | 26.12 | ND | 0.8 |  |
| Resorcinol | ND | 0.80 | 00 |  |
| Rutin | ND | ND | 15.00 |  |
| Quercetin | ND | ND | 7.00 |  |
| Kaempferol | ND | ND | 50.00 |  |
| Apigenin | ND | ND | 10.00 |  |
| Phenanthrene | ND | 0.40 | 3.00 |  |
| Pyrocatechol | ND | 0.40 | ND |  |
| Coumaric acid | 2.24 | ND | 50.00 |  |
| Ferulic acid | 7.89 | 0.20 | 0.10 |  |
| Cinnamic acid | 4.50 | 2.90 | 0.50 |  |
| Diosmin | ND | ND | 30.00 |  |
| Quinic | ND | 13.7 | 5.00 |  |

**CFM= Concentrate feed mixture consisted of corn 55%, soybean meal 8%, wheat bran 24%, cotton meal 10%, lime stone 1.2%, salt 0.8%, Sodium bicarbonate 0.3%, Vitamins and trace minerals 0.3%, Antitoxins 0.2%, live yeast 0.2%.

DM=Dry matter; CP=Crude protein; EE=Ether extract; CF=crude fiber; NFE=Nitrogen-free extract; NDF=Neutral Detergent Fiber, ND= Not detect

**Supplementary Table 2: Effect of supplementation on feed intake, digestibility of nutrients, and rumen fermentation of lactating goats.**

|  | **Control** | **F15** | **F30** | **SEM** | **p- value** |
| --- | --- | --- | --- | --- | --- |
| **Animal weight, kg** | 24.23 | 24.25 | 24.18 | 0.84 | 1 |
| **Hay intake, g/kg^0.75^** | | | | | |
| **DMI** | 22.15 | 22.10 | 22.18 | 0.19 | 0.92 |
| **EEI** | 1.37 | 1.37 | 1.38 | 0.01 | 0.89 |
| **CPI** | 3.46 | 3.45 | 3.46 | 0.03 | 0.89 |
| **NDFI** | 12.18 | 12.15 | 12.20 | 0.10 | 0.92 |
| **Total intake, g/kg^0.75^** | | | | | |
| **DMI** | 77.53 | 77.35 | 77.64 | 0.65 | 0.97 |
| **EEI** | 6.09 | 6.08 | 6.10 | 0.05 | 0.97 |
| **CPI** | 10.87 | 10.85 | 10.89 | 0.09 | 0.97 |
| **NDFI** | 28.80 | 28.73 | 28.84 | 0.24 | 0.96 |
| **Dried Fenugreek sprouts** | 0 | 1.4 | 2.75 | ND | ND |
| **Rumen fermentation** | | | | | |
| **pH** | 6.15^b^ | 5.96^a^ | 6.00^a^ | 0.03 | 0.03 |

DMI= Dry matter intake; EEI= Ether extract intake; CPI= Crude protein intake; NDFI= Neutral detergent fiber intake; ^a,b,c,d^ Means within a row with different subscripts differ significantly (p < 0.05). SEM= Standard error of means. C= control diet without supplementation; F15= control diet supplemented with dried Fenugreek sprouts at 15 g/head/day; F30= control diet supplemented with dried Fenugreek sprouts at 30 g/head/day.
